# Hair Cortisol Concentration as a Biomarker of Sleep Quality and Related Disorders

**DOI:** 10.3390/life11020081

**Published:** 2021-01-22

**Authors:** Nisrin El Mlili, Hanan Ahabrach, Omar Cauli

**Affiliations:** 1 Higher Institute of Nursing Professions and Health Techniques (ISPITS), 93000 Tetouan, Morocco; bioniss@hotmail.com (N.E.M.); hananahabrach@yahoo.fr (H.A.); 2 Department of Biology and Health, Faculty of Sciences, University Abdelmalek Essâadi, 93000 Tetouan, Morocco; 3 Department of Nursing, University of Valencia, 46010 Valencia, Spain; 4 Frailty and Cognitive Impairment Group (FROG), University of Valencia, 46010 Valencia, Spain; 5 Chair of Healthy, Active and Participatory Aging, Valencia City Council, University of Valencia, 46010 Valencia, Spain

**Keywords:** children, insomnia, REM sleep, circadian rhythm, shift work

## Abstract

Cortisol is the end product of the hypothalamic-pituitary-adrenal (HPA) axis, and its production is increased mainly in stressful situations or in chronic disorders accompanied by stress enhancement. Altered cortisol concentrations have been reported in a number of neuropsychiatric diseases and sleep disorders. Cortisol concentrations have been measured using several methods, and in several matrixes, such as blood, saliva, and urine. However, lately, hair cortisol, for several reasons, has emerged as a promising biomarker of long-term retrospective HPA activation. Several experimental approaches for cortisol measurement with the corresponding concentration reference ranges and a summary of findings from scientific literature on this field are presented. There is evidence of a close relationship between HPA functional alteration and the development of neuropsychiatric disorders. Sleep disorders are the most common manifestation in several neuropsychiatric conditions, and have also been associated to cortisol alterations in both adults and children. Many studies indicate that hair cortisol constitutes a valuable tool for further contributing to existing data on salivary, plasma, or urinary cortisol concentrations in patients with sleep disorders.

## 1. Introduction

Cortisol is the end product of the hypothalamic-pituitary-adrenal (HPA) axis. It is mainly secreted in reaction to stress, and plays a key role in normal physiology [1,2,3]. Alterations of the HPA axis and cortisol concentrations have been related to the pathophysiology of a number of diseases, including neuropsychiatric disorders [4,5,6]. The two most widely known disorders due to alterations of the HPA axis, namely Cushing’s syndrome (CS) (chronic hypercortisolemia) and Addison’s syndrome (AS) (chronic hypocortisolemia), have been reported to exhibit certain neuropsychiatric manifestations—even if not too frequently—such as depression, sleep disorders, and cognitive impairment [4,5,7,8,9,10]. In CS patients with depression and anxiety, the treatment of hypercortisolemia has been found to induce a significant decrease in the psychopathological symptoms. These results suggest a relationship between elevated cortisol and psychopathological disorders [11].

In fact, altered cortisol concentrations have been reported in a number of diseases of the central nervous system, such as psychiatric disorders, e.g., posttraumatic stress disorder [12,13,14], schizophrenia [15], depression [16,17,18,19], anxiety [20,21,22], and cognitive impairment [23,24,25]. Changes in the peripheral levels of cortisol have also been reported in certain neurodegenerative conditions, such as Huntington’s disease [26] or Alzheimer’s disease [27]. Sleep disorders are a group of conditions that affect the ability to sleep well on a regular basis, whether they are caused by a health problem or by too much stress, sleep disorders are becoming increasingly common and cortisol alterations have been linked to sleep disorders [28,29,30,31].

In addition, sleep disorders are the most common manifestation in several neuropsychiatric diseases [32,33,34] and in many other disease conditions [35,36]. Sleep disorders have been associated to alterations of the HPA axis, and cortisol levels have been correlated to several aspects of sleep disorders in patients [35,36].

Glucocorticoids are commonly used as stress biomarkers. In this regard, cortisol is the most common glucocorticoid in humans, in many mammals and in non-human primates, while in other vertebrates, such as rodents, corticosterone is the main stress hormone [1]. Although the role of cortisol in stress processes in both animals and humans is well known, data on its ability to reflect stress levels over long periods of time have been limited. This is largely due to the traditional criteria used for sample selection. Until recently, most studies investigated cortisol response from samples of saliva, serum, or urine. Both saliva and serum provide a measure of cortisol concentration at a very specific timepoint. Such samples therefore can be used to test for acute changes, but both techniques are subject to large daily physiological fluctuations—meaning that full assessment of prolonged systemic exposure to cortisol is extremely difficult. In healthy individuals, cortisol levels peak in the morning, and gradually decrease in the course of the day.

A single measurement in blood or saliva thus cannot fully reflect systemic exposure. The only solution is to obtain multiple samples throughout the waking period of the individual. Although cortisol determination in saliva has undeniable advantages over plasma, as it is a noninvasive method that does not generate additional stress, research using this method suggests that its relevance as a biological marker is applicable more to acute stress situations than to chronic processes [37]. For example, cortisol in saliva exhibited homogeneous increases in a sample of students before an oral examination, but not before a written examination [38]. However, the detection period is only 24 h, and the analysis of serum cortisol, in addition to having the same time limitations, involves an invasive technique, which in itself can cause stress—thus modifying the amount of cortisol and giving rise to an increased number of false positive results. On the other hand, plasma or serum cortisol reflects the amounts of total cortisol and not free cortisol, which is the biologically active form of the molecule [38]. For these reasons, the cortisol level in blood or urine is primarily used to diagnose Cushing’s syndrome or Addison’s disease, both of which affect the production of cortisol by the adrenal glands and the corresponding increase in cortisol concentration in urine in these endocrinology disorders are observed at any time of the day and usually lack of daily rhythm. Plasma and saliva are point samples, while urine allows the detection of recent HPA activity over a period of a few hours (24–36 h). Furthermore, cortisol concentrations in these matrixes are influenced by circadian rhythmicity or other factors that induce short-term elevations in activity of the HPA axis [39,40].

Hypercortisolism, hypocortisolism, and a blunted diurnal cortisol slope have all been associated with adverse measures of mental and physical health [1]. There is a high correlation between salivary cortisol levels and unbound cortisol in plasma and serum, which remains high during the circadian cycle and under different dynamic tests, such as adrenocorticotropic hormone (ACTH) stimulation [39,40]. Since free cortisol represents the biologically active hormone fraction, salivary cortisol measures have early been considered as a better method than serum cortisol for the evaluation of adrenocortical function; however, cortisol measurement in blood and saliva samples should be performed at different timepoints within the same day because of cortisol circadian rhythm, and it is recommended to repeat cortisol measurements in blood and saliva samples in different days since many factors can acutely influence cortisol secretion [23,24,25,39,40]. The role of hypercortisolemia in the pathophysiology of sleep disorders and neuro-psychiatric disorders accompanied by sleep alterations can only be demonstrated through measures that reflect prolonged exposure to cortisol, since such disorders are typically chronic conditions. Hair analysis has been used for decades to monitor exposure to exogenous substances in forensic science and toxicology [41]. Hair is therefore valued for its ability to reflect long periods of time with retrospective information on exposure to toxic substances [41]. It has the advantages of detection over several months, noninvasive sampling, easy storage and great resistance to decomposition—unlike body fluids, for example. For all of these reasons, hair analysis has also been introduced in the field of psychobiology. Recently, cortisol analysis in hair segments has been introduced in biomedical research, showing reliable performance as a biological marker reflecting long periods of exposure to stress, and thus extending the 24-h periods available so far [42]. The question of how cortisol is incorporated to hair has not been fully clarified, though several potential mechanisms have been suggested. In this respect, cortisol diffusion from follicular capillaries into the medulla of the hair shaft during growth [43] is the most widely accepted explanation, due to its low molecular weight and lipophilic nature that make it easy for the molecule to enter the cells. As a result, the cortisol deposited into growing hair reflects cortisol production under central HPA control.

Furthermore, the presence of a local HPA-like pathway in the hair follicle has been suggested, leading to cortisol production [44], based on studies in human scalp follicles cultured in vitro that have identified autonomous cortisol synthesis. Similarly, another study [45] recorded local cortisol production by hair follicles in response to cold (the cold pressor test), independently of the central HPA axis. However, the low hair cortisol concentration (HCC) levels found in patients with Addison’s disease suggest that the overall contribution of this alternative source to total HCC may be small [45].

Diffusion from sebum and sweat secretions into the hair follicle after formation has also been suggested [46]. The deposition of cortisol on hair from environmental exogenous sources is also possible (normally removed in a washing step of the experimental cortisol extraction procedure), though it remains unclear whether this external cortisol is incorporated into the shaft.

In the same way as cortisol measurements in saliva, cortisol concentrations in hair reflect levels of free cortisol [47], though the measurement of cortisol in hair moreover affords a reliable long-term index (of the order of months) of the activity of the HPA axis and of the systemic response to chronic stressors. The need for retrospective information about the activity of the HPA axis over long periods of time (weeks to months), and definition of the protocol for cortisol assessment in human hair samples [48], have caused hair to become increasingly interesting as a noninvasive sample material both for clinical diagnostic and research purposes.

Several advantages encourage the use of hair samples to assess cortisol concentration: (a) hair offers retrospective information about the accumulation of cortisol, allowing the measurement of chronic stress over a long term. Cortisol production in response to a stressor could be examined later in time without needing to collect the sample in the moment when stress is actually occurring. The mean hair growth rate averages about 1 cm/month [49]. Accordingly, 1 cm of hair from the scalp may represent the cortisol accumulation of one month, 2 cm of hair from the scalp may represent the cortisol accumulation of two months, and so on; (b) baseline cortisol accumulation may be recorded; samples could be extracted in the period during which stress has not yet occurred; (c) sampling is noninvasive and painless; the stress of venous sample extraction therefore can be avoided; and (d) sampling is easy, with no need for high temperature or special conditions of storage, since hair samples are characterized by great stability [41,43]. The role of diet and nutritional status, body mass index, pollution and drugs on cortisol accumulation in hair should also need a careful analysis in order to shed new lights on possible confounding factors in its determination and data interpretation.

The present study provides an overview of the methodological aspects of hair cortisol measurement and the correlations with cortisol levels measured in other matrices. We also provided an updated summary on the studies aimed to evaluate the relationship with hair cortisol measurement in association with sleep disorders in both adults and children. All original articles retrieved in the PubMed/Medline electronic bibliographic database up until 30 September 2020, were analyzed. The bibliographic searches were performed with no time limitation in each database in order to retrieve all relevant references for human studies, with the following inclusion criteria applied: (1) full text in English; (2) primary articles only; and (3) identification of data regarding measurement of cortisol measurement in hair and the analysis of hair cortisol with sleep quality and/or sleep disorders. The title and abstract were analyzed to determine which articles to include. The full text was retrieved for those that fulfilled the inclusion criteria. Finally, the reference lists of all the relevant articles were manually cross-referenced to identify any additional articles. The primary search terms used were “hair”, “cortisol” AND “sleep”.

## 2. Experimental Approaches to Cortisol Analysis

Several experimental approaches have described the way to extract and analyze hair cortisol and its concentration. In general, the main methodological steps comprise a hair sampling process, hair cutting, or pulverization, and cortisol extraction in an organic solvent. The extraction medium is then evaporated to dryness and reconstituted in saline solution or distilled water [4,42,50].

Balagova and Jezova [51] underscore that close attention must be paid to many methodological details, including the washing method, the timing and extent of sample preparation, and concrete aspects referred to the centrifugation conditions. These authors were able to optimize the cortisol extraction procedure, modifying the pulverization, methanol extraction and centrifugation steps.

The quantity of hair needed for this process is usually between 8 and 50 mg [4,42,50], and the segment lengths depend on the period of interest to be examined. Nevertheless, cortisol analysis is generally limited to the 6 cm (equivalent to 6 months) near to the scalp, because some studies have found hair extremities to be more vulnerable to degradation, and the cortisol concentrations decline as the segments lie further away from the scalp [42]. Eighteen-month hair cortisol concentrations have been assessed in some studies, however [27,28]. It is advisable to obtain the hair sample from the posterior vertex of the head, which is considered to be the region in which hair growth is more stable and exhibits less intra-individual variation [50,52].

Solid-phase enzyme-linked immunosorbent assay (ELISA), as well as luminescence immunoassay, LIA and radioimmunoassay (RIA) and liquid chromatography-mass spectroscopy (HPLC or LC-MS/MS) have been used to measure the concentration of cortisol in hair extracts [42,48,53,54,55,56]. However, because of their simplicity and low cost, ELISA kits remain the most common method used [56], even if the commercially available kits are designed for cortisol measurement in saliva rather than hair extracts [56]. Slominski et al. [57] reported that ELISA-based assays afford greater sensitivity in measuring hair cortisol levels than LC-MS-based assays. Russel et al. [58] compared four immunoassay methods and two liquid chromatography–mass spectrometry (LC-MS/MS) techniques used by four leading laboratories in hair cortisol testing, and found the results to be strongly and positively inter-correlated when analyzing a common batch of hair. Thus, laboratories using immunoassays can use a correction factor to convert the results into standard LC-MS/MS equivalents.

Careful interpretation is required when measuring hair cortisol concentration, because several confounding factors may intervene, such as radiation exposure [59], extensive washing and brushing [52,60], and chemical treatments, such as shampoos and dyes [61,62]. However, natural hair color does not seem to influence hair cortisol concentration [48,52]. Furthermore, subject age and sex [25,63], physical exercise [64], and the presence of certain comorbidities, such as diabetes and cardiovascular disorders [65] should be taken into account when measuring hair cortisol [64].

## 3. Correlation between Cortisol in Hair and in Body Fluids Used in Biomarker Analysis

To confirm the validity of scalp hair cortisol as a biological marker of retrospective systemic levels of cortisol, analyses have been made of the correlations of hair cortisol concentration (HCC) with cortisol extracted from other biological samples. It was predictable and not surprising that cortisol in hair does not correlate strongly with cortisol in other body matrices, since the latter are mainly collected only once or a few times to measure short-term cortisol exposure, while hair reflects long-term exposure. On the other hand, samples collection over a relatively long period of time may mask daily cortisol fluctuations and, thus, presumably afford a better correlation. No statistically significant correlations have been found between HCC and cortisol in one-time (point) samples of morning blood serum [52] and blood serum collected after an overnight fast [66]. In comparison, weak to moderate correlations were found with urinary cortisol.

Sauvé et al. [52] found a low yet significant correlation between hair cortisol levels and cortisol in 24-h urine, but not with serum or salivary cortisol obtained at a single time point. However, van Ockenburg et al. [67] reported a moderate and nonsignificant correlation between hair cortisol concentration and 24-h urinary cortisol concentration, even when collected over a period of 63 days. In turn, Short et al. [68] observed no correlation between cumulative one-month cortisol production in hair and the average of four weekly 24-h urinary free cortisol (UFC) determinations. Cortisol conversion or degradation could explain the lack of an association to cortisol levels in urine.

Regarding correlations with saliva, HCC was found to be moderately correlated with the mean salivary cortisol concentrations taken on three days (samples were collected at 6 timepoints on each day) [69]. Xie et al. [70] found a significant correlation between HCC in 1-cm hair segments and average salivary cortisol corresponding to three samplings (at 1, 2, and 3 weeks). The relevant study by D’Anna-Hernandez and collaborators [71] showed that cortisol concentration in hair and saliva increases throughout pregnancy, and decreases in the postpartum period. Thus, hair samples collected during pregnancy allow access to HPA activity in the first trimester, a time that was previously difficult to obtain because women may not realize they are pregnant or delay medical care until the end of the first or beginning of the second trimester, when they could be recruited for a study. Prenatal cortisol levels in the hair showed a previously verified pattern unique to pregnancy that suggests that cortisol in the hair is a convincing reflection of cortisol release over a three-month period. The high cortisol levels in the third trimester were replicated in hair, serum and saliva. The correlation of cortisol levels in hair and saliva suggests that hair cortisol levels are a useful marker of overall maternal HPA activity during each trimester. Vanaelst et al. [72] also found HCC to be significantly correlated with the salivary cortisol output (area under the curve (AUC)) of salivary cortisol collected over two consecutive days (samples were taken at four timepoints on each day). The HCC of daughters was found to be positively related to their AUC following social stress in a study of HCC as a marker of psychosocial stress in mother–daughter dyads [73]. Similarly, Kao et al. [74], in children, recorded a correlation between HCC and the AUC of salivary cortisol production in response to stress. Short et al. [68] examined the correlation between HCC and cortisol from saliva collected over the same period of one month. They found HCC to be most strongly associated with the salivary cortisol AUC over the whole month. These authors observed that the magnitude of the correlations of HCC with salivary cortisol AUC increased steadily with progressing time. Using weekly average AUC scores, summed to reflect the accumulation of time, the magnitude of the associations increased from the prior week to the prior two weeks, the prior three weeks, and the prior four weeks to the whole 30-days period. They also confirmed that HCC reflects cumulative cortisol production over a period rather than the diurnal rhythmicity of cortisol secretion.

Few studies [63,69,75] have used fingernails as a matrix to measure cortisol, due to inter-individual variability of the growth rate, and the difficulty of obtaining a sufficient sample. Izawa et al. [75] found the cortisol level in saliva over the whole day (AUC for cortisol) to be moderately associated with the cortisol level measured in fingernail samples that were collected four months and 5 months later.

## 4. Cortisol and Sleep

Sleep is a basic human need and a pillar of health [76], and is very necessary for the neurological development of infants and children. In addition, it is essential for good quality of life, better productivity, and general well-being [77].

Insufficient sleep or sleep disturbances have adverse effects upon health [78,79] and have been associated with poor physical, mental and cognitive functioning [80,81], including insulin resistance, obesity, arterial hypertension, poor cardiovascular health [79,82,83,84,85], depression, suicidal behavior, and other self-injurious behaviors [84,86,87,88], as well as overall mortality [89,90]. Sleep disruption is associated with poor mental health [91], both as a cause [92,93,94,95] and consequence [96,97,98,99].

Growing evidence suggests stress and its related physiological changes to constitute a possible link between poor sleep and adverse health effects. In particular, results from animal and human studies indicate bidirectional effects between sleep and the HPA axis [100,101].

Several studies have revealed that poor sleep is directly associated with increased basal activity of the HPA axis, and it has further been suggested that poor sleep may potentiate the responsiveness of this system to menace and defiance [100,102,103]. This possibility has been supported by a study [104] reporting strong relationships between poor sleep quality and subsequent dysregulation of the cortisol response to various stressors. Especially, poor sleep quality has been shown to predict exaggerated cortisol responses to both physiological [105] and psychological stressors [106]. It is worthy to note that repeated or chronic activation of the HPA axis may cause dysfunctions of the latter [107]. These dysfunctions may be preceded by changes in acute HPA reactivity patterns, which could be useful as early warning signs of elevated health risks. Indeed, HPA axis dysfunctions have consistently been shown to have an impact upon physiological and mental health [108], thus presenting a pathway contributing to the effects of poor sleep upon health [109].

Cortisol being the end product of the HPA axis may reflect activation of the neuroendocrine system; for this reason, it is largely used as a marker of neuroendocrine response to stress both in children and in adults [110,111].

Cortisol concentrations have been measured in several biological matrices such as saliva, urine, blood and, more recently, hair. The use of this latter matrix is an emerging method for measuring early childhood (12–60 months of age) persistent stress, and is a promising strategy for assessing chronic physiological stress responses in adults [52,112]. Hair cortisol concentration is increasingly used as a marker of HPA axis activity particularly in psycho-neuroendocrine research [113].

### 4.1. Hair Cortisol and Sleep in Adults

As already mentioned, sleep displays a close and reciprocal relationship with the functioning of the HPA axis [101,103,104,114]. Several lines of evidences have shown that stress-inducing factors can have a significant impact upon the sleep–wake cycle in various ways, depending mainly on the type of stressors and the duration of exposure (acute or chronic), as well as on inter-individual differences [115,116,117,118]. Increased stress reactivity of the HPA axis to physical and psychosocial stressors has been evidenced in people with poor quality sleep [119]. Chronic stressors profoundly impact upon human sleep architecture, as have been reported in cases of marital separation [120], burnout patients [121], or shift work [118]. In addition, higher levels of sleepiness, more frequent awakenings, lesser sleep efficiency and disturbed sleep quality have been reported in individuals with high burnout [122,123]. The characteristic of the studies evaluating hair cortisol concentration in adults and its relationship with sleep quality are shown in Table 1.

Wang et al. [124] examined the role of HPA axis activity in female employees of hospitals, and found both burnout and insomnia to be associated with elevated HCC. Likewise, burnout and insomnia have been shown to be significantly and bi-directionally associated, and mutually influence each other.

In general, strong activation of the HPA axis can lead to shortened sleep time, decreased slow-wave sleep (SWS) and sleep fragmentation [100], while insufficient sleep promotes basal activity of the HPA axis [103,104]. Animal studies have further revealed that chronic sleep disorders can induce fundamental neuroendocrine changes that may eventually influence HPA stress reactivity [103].

The quality of sleep is found to particularly influence cortisol stress responsiveness. This relates to both subjectively reported sleep quality and objectively measured sleep efficiency, awakenings during the night and time spent in lighter sleep phases. In contrast, normal variations in sleep duration do not seem to influence HPA reactivity, whereas daytime sleepiness is associated with blunted cortisol stress responsiveness [119].

The relationship between sleep and HPA axis activity has most often been assessed based on the cortisol awakening response (CAR) or the rise in cortisol measured approximately 30 min after awakening [125]. An increase in CAR has also been shown to follow poor sleep [126], though not all studies report this association [125]. A study involving postmenopausal women reported different associations between sleep quality indices and altered diurnal cortisol rhythms [127]. It has also been reported that in normal subjects, plasma cortisol levels correlate positively with induced sleep disruption [128] and negatively with total sleep time [129].

By measuring the correlation between the concentration of cortisol in saliva and sleep disturbances, participants who had a decrease of 50% or more in sleep duration showed a more marked increase in CAR compared to those who never reported sleep problems [130].

Another study in Korean firefighters using serum cortisol indicated that the serum cortisol response was positively related to nightshift work, and the serum cortisol levels differed according to the shift schedule involved [131]. Janssens et al. [132] showed that employees working a fixed day shift have significantly higher HCC levels than those involved in variable shift work.

The relationship between hair cortisol and sleep disorders has not been investigated in depth. A recent study has suggested HCC as a biomarker for sleep disorders caused by shift work [133]. The authors reported that workers with high HCC had a higher prevalence of sleep disorders compared with workers with low and intermediate HCC levels [133]. Similarly, in aneurysmal subarachnoid hemorrhage (aSAH) patients, higher HCC levels were significantly associated with increased sleep complaints [134].

In turn, a study by Feller et al. [135] suggested that daytime sleeping is positively related to higher hair cortisol levels in older individuals. In contrast, a recent study published by Lanfear and Voegel et al. [136] in older adults recorded no association between daytime sleeping and hair cortisol.

These controversial results could be due to the differences in sleep duration. Since endogenous cortisol levels increase shortly after waking, it would be expected that the HCC levels would be higher in adults who are sleeping during the day for a long period, and not in individuals who sleep for only a very short time [137].

### 4.2. Hair Cortisol and Sleep in Children

The activity of the HPA axis is typically assessed by measuring cortisol as the end product of the axis [138]. The variation in diurnal cortisol secretion patterns has been linked to sleep behavior [106,137,138,139]. These associations may be especially important for understanding the mechanisms underlying these bidirectional associations during the pre- and post-adolescent years when these subjects are experiencing significant physical, neurobiological, and socioemotional changes [140,141].

It has been reported that chronic sleep difficulties and insufficient sleep experienced during childhood independently result in the development of later physical and mental health problems in many young individuals [142,143]. Several epidemiological studies have reported that up to 50% of all children experience sleep problems [144,145,146], often having poor sleep health, including insufficient sleep duration and/or poor sleep efficiency [147,148]. Additionally, insufficient sleep has been related to multiple problems, such as an increase in risk-taking behaviors [149] and a decline in school performance [150].

The relationships between sleep and the HPA axis have not been widely investigated in children. The few studies that have examined sleep and basal salivary cortisol in infancy support the sensitivity of the infant HPA axis to sleep [151,152]. Indeed, in infants, higher waking salivary cortisol concentration (SCC) and the cortisol awakening response (CAR) were associated with taking more daytime naps, and were positively related to later sleep onset [151]. Likewise, in infants between 2 and 4 years of age, lower waking SCC the next morning was associated with forced sleep restriction, and CAR was associated with day-time naps [152].

Other studies have reported that children with short and poor sleep present increased HPA axis activity after awakening [106,153,154]. Increased morning cortisol secretion in children with short sleep duration, shorter relative amounts of slow-wave sleep (SWS), longer relative amounts of light sleep (including stage 1 sleep and stage 2 sleep), and rapid-eye-movement (REM) sleep have been reported [155,156].

Importantly, there is evidence that sleep quality (nocturnal awakening, disordered breathing, SWS) is affected in children born very preterm in comparison with term born peers [157,158], which may lead to sleep disturbances during adolescence and young adulthood, with earlier bedtimes and circadian preferences [159,160,161]. Recent studies have examined the relationship between hair cortisol and sleep in children (Table 2).

Although little is known about the potential long-term health effects of elevated hair cortisol in children, a study conducted in children born into families affected by multiple adverse psychosocial exposures found that such children seemed to have higher cortisol levels and were significantly more affected by the most common childhood disease conditions [162]. In the same sense, Vanaelst and al. [66] reported a positive correlation between HCC and major childhood life events in children.

A study assessing the relationship between hair cortisol and sleep duration in 12-month old infants reported that HCC in infants who slept 10 h or more each night were lower than in infants who slept less than 10 h [163]. However, a recent study examining the association between sleep quality and quantity and chronic stress levels of hair cortisol in children aged 2–6 years reported no association between sleep and cortisol levels [164].

Nevertheless, authors that have examined the association between sleep quality and salivary cortisol levels among children seem to generally agree that shorter sleep duration and/or longer sleep onset latency is directly related to cortisol measured in saliva, in both longitudinal and cross-sectional studies. Findings from several studies in children have reported direct associations between salivary cortisol levels and poor sleep habits, sleep patterns, and sleep problems, including shorter sleep duration and poorer sleep quality [155,156,165,166].

Childhood HPA function is complex, and various measures of cortisol in children are essential to allow comprehensive understanding of its environmental correlates and long-term implications. In this regard, research into the association between hair cortisol and sleep is still in its beginnings, and future long-term studies affording more detailed information on objectively measured sleep are still needed to determine whether high hair cortisol may influence sleep characteristics in children.

In sum, sleep plays an important role in maintaining adequate neuroendocrine stress reactivity, and inadequate sleep potentiates the stress reactivity of the HPA axis [119]. Evidence from experimental studies has established that sleep affects HPA function [109,167,168]. Accordingly, impaired sleep increases the activity of the HPA axis [103,104], and elevated levels of HPA hormones are found to result in inadequate sleep [169,170,171]. Although the relationship between sleep and the HPA axis is clearly bidirectional, these findings support the assumption that sleep specifically modulates the stress sensitivity of the HPA axis.

## 5. Conclusions

Cortisol alterations seem to be intimately associated with sleep disorder and the development of neuropsychiatric disorders and symptoms. Sleep disorders associated with high cortisol levels in both adults and children. However, we need studies to assess whether hair cortisol can also reflect the effects of pharmacological and non-pharmacological interventions aimed to improve sleep quality. The measurement of hair cortisol and its relationship with more “acute” measurements, e.g., saliva and blood levels is particularly warranted in patients with severe cognitive impairment in which clinical assessment of sleep complaints is difficult or impossible. The role of diet and nutritional status, body mass index, pollution, and drugs on cortisol accumulation in hair should also need a careful analysis in order to shed new lights on possible confounding factors in its determination and data interpretation. Many studies indicate that hair cortisol constitutes a valuable tool to further supplement existing data on salivary, plasma, or urinary cortisol concentrations in patients with sleep disorders, and future studies would validate hair cortisol determinations as diagnostic and prognostic tool in several neuropsychiatric and endocrine disorders.

## Figures and Tables

**Table 1:** Hair cortisol and sleep disorders in adults.

Reference	Number of Adults and Mean Age	Characteristics of Participants	How the Analysis of Sleep was Performed	Mean Concentration of Hair Cortisol (HCC)	Main Findings of the Study Related to Hair Cortisol Concentration
Lanfear et al., 2020	N = 42 Age: 60–80 years Mean age: 68.1 ± 5.3 years.	Male and female over the age of 60 years; older adults in good general physical health. Participants were divided into two categories based on whether they did or did not sleep during the day.	An ad hoc questionnaire to capture physiological data: time and duration of daytime sleep.	HCC levels: 1.4–82.5 pg/mg Median HCC: 5.65 pg/mg Mean HCC: 10.5 pg/mg ± 13.6 pg/mg Mean HCC in males (n = 20) was 14.4 pg/mg Mean HCC in females (n = 22) was 6.8 pg/mg	HCC in older adults trends to be higher in comparison with toddlers and adolescents. A significant difference in hair cortisol between males and females older adults. No significant difference in hair cortisol between participants who did and did not sleep during the day.
Trikojat et al, 2017	Season Allergic Rhinitis (SAR) patients (n = 41) Age mean: 24.12 (±3.3) years Healthy controls (n = 42) Age mean: 24.43 (±3.1) years.	SAR patients reported rhinitis symptoms exclusively during pollen season and had a positive skin prick test for at least one grass or tree pollen and/or elevated total IgE level (>100 IU/mL serum). All patients were off allergy medication for at least seven days prior to testing.	Pittsburgh Sleep Quality Index (PSQI; Buysse et al., 1989).	Hair Cortisol, pg/mg SAR patients: On-pollen season 5.4 (±4.3) Off-pollen season 4.2 (±2.5) Healthy controls: On-pollen season 5.9 (±4.2) Off-pollen season 4.1 (±2.6)	No significant group differences in HCC could be found in both testing seasons. On allergy season, Beck Depression Inventory II sum score correlates with PSQI but not with HCC in SAR patients.
Zhang et al, 2020	N = 435: men = 164 women = 271 Age range (20–58) years, Average age (38.32 ± 7.42) years.	Participants are classified as: Fixed day workers: 127 (29.20%) Two-shift workers: 87 (20%) Three-shift workers: 64 (14.71%) Four-shift workers: 135 (31.03%) Other workers: 22 (5.06%)	The Pittsburgh Sleep Quality Index scale PSQI; Buysse et al., 1989)	HCC range: 1.07–9.67 ng/g hair HCC median: 3.20 (2.15–4.71) ng/g hair HCCs were dichotomized at Q3 (4.71 ng/g hair) threshold	HCC levels of workers with different shift patterns were significantly higher than that of fixed day shift workers. Compared with workers with low and intermediate HCC, the prevalence of sleep disorders in workers with high HCC was significantly higher.
Wang et al., 2019	N = 68 females Age range: 22–51 years (32.50 ± 6.13),	Participants were employees working in both clinical and management roles, who had worked continuously in the same position for at least 6 months, and Participants had no history of mental illness and no history of psychotropic drug use for 1 week prior to the investigation, basing on the clinical records and self-report of each participant.	Athens Insomnia Scale (AIS),	HCC median: 5.89 ng/g hair Interquartile HCC range: 2.20–10.74 ng/g hair	HCC was significantly associated with insomnia. No significant mediating effect of HCC on the path from insomnia to burnout. Both burnout and insomnia were associated with elevated HCC among female employees of hospital.
Colledge et al., 2017	Patients with aneurysmal subarachnoid hemorrhage (aSAH) N = 32: 22 women, 10 men; Mean age: 57.4 ± 10.7 years; Healthy controls N= 17: 11 women, 6 men; Mean age = 59.8 ± 10.8 years	Patients with aSAH following neurosurgical or endovascular intervention.	The 7-item Insomnia Severity Index (Bastien CH, Vallieres A, Morin CM, 2001)	HCC mean: aSAH patients: 4.24 ± 2.91 nmol/L, Healthy controls: 2.39 ± 1.26 nmol/L	HCCs were significantly higher in aSAH patients compared to healthy controls. In aSAH patients, higher HCCs were significantly associated with increased sleep complaints. aSAH patients exhibited more sleep complaints.
Feller et al., 2014	N = 680 participants (369 women) Mean age: 65.8 years Range age: 47–82 years.	Participants in middle and old adulthood not using glucocorticoid-containing treatments.	Average night and daytime sleep was collected using questionnaires	Mean ± SD HCC in the whole sample were 35.1 ± 32.8 pg/mg. Mean ± SD SCC were 37.9 ± 34.0 pg/mg in men and 32.7 ± 31.6 pg/mg in women. HCC outlying by 3 standard deviations (SD) from the mean were excluded (n = 18) after the initial elimination of extreme values >500 pg/mg (n = 8), resulting in a final sample of 654 participants for further analyses.	A linear increase in HCC with participant ages was found and this relationship was maintained regardless of the influence of other age-related variables (body mass, physical activity, alcohol consumption, smoking). HCC was found to be positively related daytime sleeping. In multiple regression analysis, daytime sleeping was not a predictor factor of HCC.

**Table 2:** Hair cortisol and sleep disorders in children.

Reference	Number of Children and Mean Age	Characteristics of Participants	How the Analysis of Sleep Was Performed	Mean Concentration of Hair Cortisol (HCC)	Main Findings of the Study Related to Hair Cortisol Concentration
Flom et al., 2017	Age: 12 months. Mean age: 12.18 N = 111	Healthy infants who had no known hearing, visual, neurological, or developmental disorders. Infant ethnicity: Caucasian 65.5% Asian 4.5% Black 4.5% Hispanic 1.8% Native American 0% Multiracial/Other 23.6% Infants were classified into High HCC and Low HCC Salivary Cortisol concentrations (SCC) were also measured at waking and bedtime and infants were classified to steep slope and flat slope.	The Brief Infant Sleep Questionnaire (BISQ; Sadeh, 2004) completed by mothers. Infant nighttime sleep duration: time in hours that the infant typically spends asleep between 7 p.m. and 7 a.m. Sleep disruption: number of night wakings (<2 times; >2 times per night).	HCC (pg/mg): 86.26 ± 183.63.	Infants who slept 10 h or more each night at 12 months had lower HCC than infants who slept less than 10 h. Sleep disruption was higher for infants with flat slope/high HCC compared to infants with a steep slope/high HCC. Higher infant HCC was associated with greater infant waking SCC, bedtime SCC, but not with diurnal slope. Sleep disruption was not related to infant HCC at 12 months.
Eythorsdottir et al., 2020	Age: 2–6 years. Mean age: 5.3 years. N = 68 for sleep information. N = 72 for hair cortisol measurements.	Pre-school children having factors for overweight predisposition.	Objective sleep characteristics (sleep duration, sleep latency and sleep efficiency) assessed by an ActiGraph GT3X during a continuous period of 5 days and nights.	Cortisol levels (pg/mg): 109. Cortisol levels overall median range = 7–890.	Sleep characteristics were generally not associated with log transformed cortisol levels.
Maurer et al., 2016	Healthy children born very preterm (<32nd gestational weeks): Mean age: 9.5 ± 1.4 years. Age range: 7.4–12.4 N = 85 Healthy children full term born: Mean age: 9.6 ± 1.4 years Age range: 6.9–13 N = 91	Healthy children at school age.	Sleep was assessed using in-home polysomnography (PSG) during a single night at the children’s home. Objective sleep indices were evaluated: sleep continuity, sleep efficiency and nocturnal awakening. Sleep architecture (%): Stage 1 sleep, stage 2 sleep, SWS (SWS: Stages 3 and 4 sleep), REM sleep, and REM latency (min).	Hair cortisol values (truncated to a value of 2 inter quartile above the median and adjusted for socio-demographic characteristics) Very pre-term Cortisol mean: 2.0 ± 2.1 Full-term Cortisol mean: 1.9 ± 1.8	A negative association between hair cortisone and child age; boys had significantly higher hair cortisol and cortisone levels than girls. A negative association between sleep duration and child age; boys showed longer REM sleep latency than girls. No other significant relations between sleep measures and child age.
